# Obesity in pregnant women: maternal, fetal, and transgenerational consequences

**DOI:** 10.1038/s41430-021-01015-z

**Published:** 2021-10-26

**Authors:** Alexander Strauss

**Affiliations:** Faculty of Medicine, Christian-Albrechts-University, Kiel, Germany

**Keywords:** Clinical trials, Nutrition

Obesity is no longer a problem exclusively affecting the western world. Although developing countries across the Caribbean, Middle East, and Pacific Islands have all seen a rapid increase of obesity rates. Worldwide, 11.5% of adults were obese in 2010 with nearly a tripling to 30% (2.1 billion) in 2020. Since women are an integral part of this overflowing wave, the impact of obesity on health and wellbeing of mothers and their offspring is profound. Consequently, maternal obesity (Fig. 1) challenges modern perinatology [1].

**Fig. 1:**
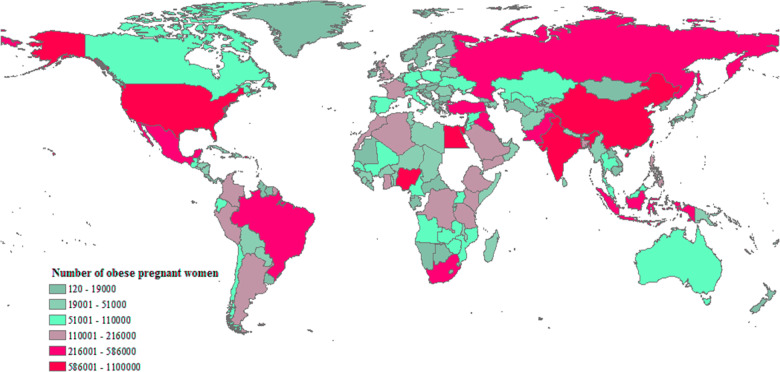
Obesity in pregnant women. The estimated distribution of obese pregnant women—the global perspective (184 countries, 2014) [1].

## Scope of the problem

Obesity is no new challenge; however, the extent of the problem today is unprecedented, and its sequelae for women’s health are undeniable. Poor weight control contributes to more deaths than smoking, alcohol and sedentary lifestyle combined, leading to 12% of all deaths (UK). Consequently, obesity also challenges modern obstetrics. Beside the medical problems, the socioeconomic impact of maternal obesity is to be faced by society as a whole. In the UK, an additional financial burden afforded by obesity on healthcare resources was £16 billion per year in 2007 with a predicted rise to £50 billion by the year 2050.

## Overcoming obstetrical obstacles

Etiology and consequences of obesity in the context of maternity are multifactorial. Since obesity is associated with adverse maternal and fetal outcome, obese mothers require a risk-adapted pregnancy management. As most of the issues are not trivial to study, quantifying is one key to tackling pending questions. Reliable scientific evidence to guide obstetricians is sustainably needed. To achieve the goals set, concerted social and medical efforts need broad collaboration to ameliorate the effects of maternal obesity on future generations.

## Obesity a disease?

In 2013, the American Medical Association supported by several US national medical specialty organizations published Resolution 420 (A-13) recognizing obesity as a disease state with multiple pathophysiological aspects requiring a range of interventions to improve its prevention and treatment [2]. The aim of this decision was to encourage a broader spectrum of healthcare benefits insurance coverage for the prevention and treatment of obesity in North America. In the meantime, this claim has become a worldwide task.

## Obesity in pregnant women: maternal, fetal, and transgenerational consequences

As obesity of mothers spreads in our societies, it is inevitable for all maternity care clinicians to face its particular challenges during pregnancy and delivery. Hence maternal body weight substantially influences the odds e.g. for impaired fertility, miscarriages, fetal anomalies, birth risks and various long-term adverse sequelae for mothers and their offspring, differentiated basic research is fundamental. This thematic issue of *EJCN* offers a forum for renowned international experts to discuss their current findings.

## (Epi-)Genetics

The knowledge of pathophysiological consequences associated with maternal obesity and its consequences on placental structure and function in relation to epigenetic mechanisms is still evolving. Emerging data indicate a putative interaction of epigenetically mediated effects of maternal obesity on weight development and cardio-metabolic risk profile in the offspring. In his work Reichetzeder, calls for further research that combines genome-wide and epigenome-wide association studies [3]. During the development of obesity, the adipose tissue adapts to the excessive lipid load (adipocyte size/number, remodeling of immune cell composition extracellular matrix and lipid metabolism). Lipotoxicity insults other organs, resulting in insulin resistance or diabetes is substantially promoted by a metabolic stress load established by the concomitant pregnancy. Considering that these process failures end up in enduring defects in adipose tissue expandability/functionality, Corrales et al. postulate an underlying epigenetic memory in adipocytes transmitted through generations [4].

## Pregnancy risks

Longitudinal analyses of mother’s anthropometric give insights on the long-term development of body weight. On the other hand, desirable maternal weight range/gain to optimize pregnancy course and outcome is still unknown [5]. On a cellular basis endocrine mechanisms (placental dysfunction) influence adipocyte differentiation and fatty acids homeostasis. A more detailed understanding of the pathological pathways influencing adipose tissue biology is examined by focusing on the impairment of adipocyte expansion. Trivett et al. observe relations to an increase of maternal insulin resistance and diabetes mellitus type 2 [6].

## Interventions

A healthy lifestyle before and during pregnancy that incorporates exercise is essential preventing excessive weight gain or the development of gestational diabetes. Therefore Ferrari et al. promote physical activity as a key tool to reduce the pro-inflammatory effects of adipose tissue [7]. Øhman et al. extend this observation by finding that diet and exercise therapies improve metabolic fitness (body composition) in obese mothers, even during lactation [8]. Meta-analytic results on the effectiveness of lifestyle intervention and bariatric surgery to reduce the risks of gestational hypertension in obese women emphasize the need to modulate weight in obese women already preconceptionally [9].

## Men

Talking about human reproduction the focus of prophylactic preconception intervention strategies needs to be revised with a shift from women to couples. Observational evidence suggests that metabolic changes due to overweight/obesity affect epigenetic markers in oocytes and sperms alike (gamete quality) and may influence epigenetic programming and reprogramming processes during embryogenesis. Therefore Hieronimus et al. delineate that fathers contribute to the health trajectory of their progeny and this needs to be a part of considerations before all-femal body weight interventions are implemented. Protection of future generation’s undesirable metabolic programming therefore warrants scientific awareness on interactions between maternal and paternal lifestyle and health status [10].

## Perspective debate

With this themed issue, we aim to highlight diversified research that spans fertility problems, antenatal/intrapartum care and postnatal health issues as well as lifestyle factors and transgenerational consequences in relation to obesity in pregnancy. Education, support of healthy choices, and targeted promotion of lifestyle interventions are urgently warranted for the benefit of this and future generations.

## References

[1] Cheng C, Xianglong X, Yan Y. Estimated global overweight and obesity burden in pregnant women based on panel data model. PLoS ONE. 2018. 10.1371/journal.pone.0202183. 10.1371/journal.pone.0202183 PMC6084991 30092099

[2] American Medical Association. Resolution 420 (A-13). 2013. https://media.npr.org/documents/2013/jun/ama-resolution-obesity.pdf.

[3] Reichetzeder C. Overweight and obesity in pregnancy: its impact on epigenetics. Eur J Clin Nutr. 2021. 10.1038/s41430-021-00905-6 PMC8636269 34230629

[4] Corrales P, Vidal-Puig A, Medina-Gómez G. Obesity and pregnancy, the perfect metabolic storm. Eur J Clin Nutr. 2021. 10.1038/s41430-021-00914-5 33911209

[5] Strauss A, Rochow N, Kunze M, Hesse V, Dudenhausen JW, Voigt M. Obesity in pregnant women: a 20-year analysis of the german experience. Eur J Clin Nutr. 2021. 10.1038/s41430-021-00981-8 PMC8636254 34702964

[6] Trivett C, Lees ZJ, Freeman DJ. Adipose tissue function in healthy pregnancy, gestational diabetes mellitus and pre-eclampsia. Eur J Clin Nutr. 2021. 10.1038/s41430-021-00948-9 PMC8636251 34131300

[7] Ferrari N, Joisten C. Impact of physical activity on course and outcome of pregnancy from pre- to postnatal. Eur J Clin Nutr. 2021. 10.1038/s41430-021-00904-7 PMC8636258 33828239

[8] Øhman EA, Kirchner L, Winkvist A, Bertz F, Holven KB, Ulven SM, et al. Effects of dietary and exercise treatments on HDL subclasses in lactating women with overweight and obesity. Eur J Clin Nutr. 2021. 10.1017/S0007114522000241 PMC9661371 35067237

[9] Schenkelaars N, Rousian M, Hoek J, Schoenmakers S, Willemsen S, Steegers-Theunissen R. Effectiveness of preconceptional maternal weight loss by lifestyle interventions or bariatric surgery and the risk reduction of hypertensive disorders of pregnancy; a systematic review and meta-analysis. Eur J Clin Nutr. 2021. 10.1038/s41430-021-00902-9 33837274

[10] Hieronimus B, Ensenauer R. Influence of maternal and paternal pre-conception overweight/obesity on offspring outcomes and strategies for prevention. Eur J Clin Nutr. 2021. 10.1038/s41430-021-00920-7 PMC8636250 34131301

